# Comparative genomics, phylogenomic reconstruction and divergence time analysis of 32 *Delphinium* species based on complete chloroplast genomes

**DOI:** 10.3389/fpls.2026.1925719

**Published:** 2026-09-03

**Authors:** Ting Lv, Yu Zhang, Lizhi Guo, Xiayu Hu, Jiasheng Ju, Nan Tang

**Affiliations:** 1 College of Agriculture and Animal Husbandry, Qinghai University, Xining, China; 2 Key Laboratory of Qinghai Province for Landscape Plants Research, Plateau Flower Research Centre, Qinghai University, Xining, China; 3 College of Agronomy, Northwest A&F University, Yangling, China; 4 Qinghai Institute for Drug Control, Xining, China; 5 Food and Drug Testing Center, Xianyang, China

**Keywords:** chloroplast genome, *Delphinium*, divergence time, phylogenomics, Qinghai-Tibetan plateau

## Abstract

*Delphinium*, characterized by distinctive spurred flowers, comprises numerous high-altitude endemic species with significant ornamental and medicinal value. However, the intrageneric phylogenetic relationships and evolutionary history of *Delphinium* have remained poorly resolved, primarily due to the limited variability of traditional DNA markers and complex morphological homoplasy. In this study, complete chloroplast genomes of three *Delphinium* spp. were sequenced and combined with those of 29 *Delphinium* spp. publicly available plastomes to conduct a comprehensive phylogenomic analysis and divergence time estimation. The *Delphinium* plastomes exhibited a highly conserved quadripartite structure, with genome sizes ranging from 153,769 to 157,339 bp. Comparative genomic analyses revealed 2,426 SSRs, and although narrow variable windows (*π* > 0.02) were detected within or adjacent to five loci (*ndhA*-*ndhH*, *ycf2*, *trnG*-*trnfM*-*rps14*, *rrn23*, *trnI*), their overall sequences remained highly conserved at the species level. Given their moderate overall divergence and reduced substitution saturation, these five loci are suitable for higher-level phylogeny, whereas conventional DNA barcodes and SSC-derived regions remain more effective for species-level discrimination. Phylogenomic reconstruction based on whole plastomes robustly supported the monophyly of *Delphinium* and resolved six well-supported primary clades, outperforming concatenated protein-coding genes in topological resolution. Furthermore, divergence time estimation revealed that the *Delphinium* crown group originated in the early Eocene (ca. 53.23 Ma; 95% HPD: 51.68-54.77 Ma). An initial major lineage divergence occurred during the Oligocene (ca. 28.53 Ma) driven by global cooling, followed by exPLoSive diversification during the Pliocene and Pleistocene (e.g., rapid radiation of core clades at 3.47 Ma). This recent rapid radiation was likely triggered by the intense uplift of the Qinghai-Tibet Plateau and recurrent Northern Hemisphere glaciations. Collectively, our study provides new insights into the plastome evolution and phylogeny of *Delphinium*, offering a robust evolutionary framework for understanding its diversification and facilitating future population genetic analyses.

## 1 Introduction

*Delphinium* is a phylogenetically significant genus within the family Ranunculaceae, comprising approximately 300–400 species worldwide with its diversity center concentrated in the Qinghai-Tibet Plateau and adjacent regions (Malyutin, 1987). *Delphinium* species are typically perennial herbs displaying substantial variation in basal foliage and inflorescence structures. The genus is morphologically distinguished by a tubular or subulate spur extending from the posterior sepal (Wang and Warnock, 2001). The specialized floral morphology of *Delphinium* represents a key evolutionary innovation underpinning its adaptive radiation, likely driven by pollinator-mediated coevolution (Sharma et al., 2024). In addition to their evolutionary significance, *Delphinium* species hold considerable economic and ecological value. They are widely cultivated as ornamentals due to their diverse floral traits, and are of ethnobotanical interest for their therapeutic properties, although the presence of toxic alkaloids (specifically diterpenoid alkaloids) necessitates cautious use (Yin and Jiang, 2023). From an ecological perspective, these species are integral to alpine meadow ecosystems, functioning as vital nectar and pollen sources that support pollinator communities (Guerrant, 1982; Qian et al., 2022). However, despite their multifaceted importance, the taxonomy and phylogeny of *Delphinium* remain unresolved and phylogenetically challenging.

Resolving phylogenetic relationships and estimating divergence times are fundamental to understanding evolutionary history, biogeographic patterns, and adaptive radiation. Traditional phylogenetic studies of *Delphinium* have predominantly relied on a limited number of nuclear (e.g., ITS) and chloroplast markers (e.g., *atpB*, *rbcL*) (Hoot, 1995). However, these markers frequently lack sufficient resolution to resolve relationships among closely related taxa, resulting in polytomies or incongruent topologies. Similarly, previous estimates of divergence times were constrained by limited taxon sampling and a paucity of genetic data, resulting in imprecise estimates for the genus’s origin and diversification (Ramírez-Rodríguez et al., 2019; Park et al., 2020). Therefore, the application of chloroplast phylogenomics provides a valuable foundational approach toward improving the phylogenetic framework for *Delphinium* and elucidating the spatiotemporal dynamics of its evolution.

The chloroplast is a semi-autonomous organelle that plays vital roles in photosynthesis and the biosynthesis of starch, fatty acids, and pigments in plants (Daniell et al., 2016). It possesses an independent genome, which is typically a double-stranded circular DNA molecule that replicates independently of the nuclear genome (Daniell et al., 2016). The angiosperm plastome typically displays a conserved quadripartite structure, consisting of a large single-copy (LSC) region and a small single-copy (SSC) region separated by two inverted repeats (IRs) (Mahai et al., 2024). While generally ranging from 120 to 160 kb in size, significant size variations exist in certain taxa, such as *Pelargonium* (Palmer et al., 1987). Due to its conserved gene content, moderate nucleotide substitution rates, and lack of recombination relative to nuclear and mitochondrial genomes, chloroplast DNA (cpDNA) has emerged as a premier tool for phylogenetic reconstruction (Daniell et al., 2016). Recently, comparative plastomics has enabled the detection of mutational hotspots, structural variations (e.g., inversions and gene rearrangements), insertions-deletions (InDels), and single nucleotide polymorphisms (SNPs), providing a wealth of informative markers for evolutionary inference (Walker et al., 2019). Additionally, the stability of the plastome makes it an ideal resource for divergence time estimation when combined with reliable fossil evidence (Magallón et al., 2015). Indeed, chloroplast phylogenomics has proven effective in resolving ambiguous phylogenetic relationships in complex lineages like *Aconitum* (Wang et al., 2024) and *Pulsatilla* (Li et al., 2020), and has simultaneously provided a robust temporal framework to elucidate their diversification history.

Recently, genomic studies have begun to elucidate the chloroplast architecture of *Delphinium*, providing a foundation for understanding the genus’s evolutionary dynamics. For instance, the *Delphinium denudatum* plastome as a typical quadripartite circle of 154,228 bp comprised 111 unique genes, with the notable deletion of the *rps16* and *rpl32* genes (Li et al., 2025). Subsequent phylogenomic investigations involving 14 plastomes have further clarified evolutionary relationships, confirming the genus’s monophyly and distinguishing between perennial and annual groups. These analyses also screened nine hypervariable regions (e.g., *ndhF*-*trnL*, *ycf1*) to serve as potential barcodes (Song et al., 2024). Furthermore, codon usage analysis in *Delphinium grandiflorum* has demonstrated a preference for AT-ending codons driven by natural selection (Duan et al., 2021). A recent phylogenomic study based on 117 plastomes provided a robust tribal-level framework for *Delphinieae* (Luo et al., 2026). However, that study was primarily designed to resolve deep suprageneric relationships, and included relatively few representatives from each major *Delphinium* subclade. Consequently, the rapid, shallow-level diversification events within the genus remain insufficiently resolved. Furthermore, although Luo et al. (2026) estimated the crown age of the *Delphinium* lineage at approximately 33.30 Ma, its true temporal origin may be considerably older, as sparse internal taxon sampling frequently leads to the underestimation of clade crown ages (Linder et al., 2005). This potential discrepancy highlights the critical need for a dedicated, infrageneric temporal framework. Importantly, the potential roles of the late Cenozoic Qinghai-Tibetan Plateau uplift and Pleistocene climatic oscillations in driving recent speciation events within *Delphinium* remain largely unexplored in a phylogenetic context. To address these gaps, we integrated publicly available genomic resources with newly sequenced data to compile a targeted phylogenomic dataset of 32 *Delphinium* species, ensuring representative coverage of the phylogenetic diversity within its major infrageneric subclades. While more focused in taxonomic scope than the tribe-scale dataset of Luo et al. (2026), our sampling provides substantially higher density at the infrageneric level. This targeted approach enables us to resolve relationships among recently diverged *Delphinium* lineages that are typically poorly resolved in higher-level analyses.

Specifically, our objectives were to: (1) identify nucleotide diversity hotspots and SSR markers to provide resources for future population genetic and phylogeographic studies; (2) reconstruct the phylogeny of the genus based on whole plastomes and protein-coding datasets; and (3) estimate divergence times using molecular clocks with secondary calibrations, and evaluate the potential correlations between the rapid diversification of *Delphinium* and late Cenozoic tectonic uplifts and paleoclimatic fluctuations. Overall, this infrageneric analysis provides a comprehensive evolutionary framework for *Delphinium*, offering deeper insights into its historical biogeography and speciation dynamics that are difficult to achieve with broad, tribe-scale phylogenies alone.

## 2 Materials and methods

### 2.1 Genomic DNA extraction and sequencing from plant materials

Based on the established morphological classification system, the genus *Delphinium* comprises three subgenera and five sections (Wang and Warnock, 2001). To reconstruct the phylogenetic framework of *Delphinium*, we assembled a dataset comprising 32 complete chloroplast genomes. This dataset included 29 sequences retrieved from NCBI and three newly sequenced species: *Delphinium densiflorum*, *D. albocoeruleum*, and *D. trichophorum*. The three sampled species were collected from Qinghai Province, China. Specifically, the detailed collection information of each species is presented as follows. *D. densiflorum* was collected from Guoshize Mountain, Xiaoqianhu Village, Garang Township, Guide County (36.29°N, 101.61°E; elevation 4,031 m; habitat: flowstone area; collection date: August 2024). *D. albocoeruleum* was collected from Shanglashou Township, Yushu City (33.00°N, 96.38°E; elevation 4,295 m; habitat: alpine meadow; collection date: July 2024). *D. trichophorum* was collected from Maqin County (34.38°N, 100.32°E; elevation 3,837 m; habitat: alpine meadow; collection date: July 2024). All voucher specimens (voucher numbers: Lv Ting 202470, Lv Ting 202438, Lv Ting 202417) were deposited in the Herbarium of Qinghai University. Subsequently, total genomic DNA was extracted from the leaf tissue using a modified CTAB method (Gawel and Jarret, 1991). The integrity and purity of the extracted DNA were evaluated by 1% agarose gel electrophoresis. High-quality DNA was fragmented by ultrasonication, and sequencing libraries were constructed following standard protocols, including end-repair, A-tailing, adapter ligation, and PCR amplification. After strict quality control, the libraries were sequenced on the Illumina NovaSeq platform (PE150) at Nanjing Genesky Biotechnologies Inc. (Nanjing, China). Raw reads were subsequently filtered to generate clean data for assembly and annotation analysis.

### 2.2 Assembly and annotation of the chloroplast genome

After the removal of low-quality reads, high-quality reads of the three species were assembled *de novo* into complete chloroplast genomes using GetOrganelle v1.7.5.0 with a k-mer setting of 55. The resulting assembly graphs were visualized and rigorously validated using Bandage v0.8.1 (Wu et al., 2007) to assess structural integrity and assembly accuracy. Genome annotation was performed using a reference-based strategy. Specifically, the assembled plastomes were annotated by transferring annotations from the reference genome of *D. brunonianum* (NC_051554.1) using Geneious Prime v8.1 (Kearse et al., 2012). The initial annotations were then manually curated to correct misannotations, remove redundancies, and accurately define start/stop codons and intron-exon boundaries. Finally, circular maps of the annotated chloroplast genomes were generated using Chloroplot software (https://irscope.shinyapps.io/Chloroplot/).

### 2.3 Comparative analyses and IR boundary analysis

To investigate structural evolution and sequence divergence within *Delphinium* species, we performed a comprehensive comparative analysis of the 32 assembled chloroplast genomes. Complete chloroplast genomes of 32 *Delphinium* species were aligned using Geneious Prime v8.1 (Kearse et al., 2012). To assess interspecific divergence and identify hypervariable regions, nucleotide diversity (*π*) was calculated using a sliding window analysis in DnaSP v6.0 (Rozas et al., 2017), while nucleotide substitutions and genetic distances (p-distance) were computed using MEGA 7.0 (Kumar et al., 2016). We systematically compared key structural features across all taxa, including total plastome size, GC content, gene counts (protein-coding, tRNA, rRNA), and the lengths of the LSC, SSC, and IR regions. Furthermore, evolutionary dynamics of the IR regions were elucidated by examining IR expansion and contraction events. The junction sites (JLB, JSB, JSA, JLA) were visualized and compared using IRscope (Amiryousefi et al., 2018) to detect boundary shifts and associated gene content variations. Simple sequence repeats (SSRs) in the chloroplast genome of 32 *Delphinium* species were detected using MISA (Androsiuk et al., 2018). The minimum repeat thresholds were set to 10, 5, 4, 3, 3, and 3 for mono-, di-, tri-, tetra-, penta-, and hexanucleotides, respectively.

### 2.4 Phylogenetic analyses

To reconstruct the phylogeny of 32 *Delphinium* species, we employed Maximum Likelihood (ML) and Bayesian Inference (BI) methods on two datasets: (i) the complete plastome sequences, and (ii) concatenated protein-coding genes (PCGs). Sequences were aligned using MAFFT v7.427 (Katoh and Standley, 2013) implemented in PhyloSuite and subsequently trimmed with trimAl (Capella-Gutiérrez et al., 2009) to remove ambiguously aligned regions. *Coptis chinensis* and *Thalictrum alpinum* were selected as outgroups. Optimal substitution models for each dataset and analysis type were independently evaluated using ModelFinder (Kalyaanamoorthy et al., 2017). ML analyses were performed in IQ-TREE v.2.1.3 (Nguyen et al., 2015) using the K81+F+I+R4 model for the complete plastome sequences and the TVM+F+I model for the PCGs, with 5,000 ultrafast bootstrap replicates. For BI analyses, model selection was specifically restricted to those implemented in MrBayes. Based on the Bayesian Information Criterion (BIC), the GTR+F+I model was independently identified as the best-fit substitution model for both datasets. Bayesian analyses were conducted in MrBayes v.3.2.7 (Ronquist et al., 2012), running for 10 million generations and sampling trees every 1,000 generations. Convergence of the Markov Chain Monte Carlo (MCMC) runs was confirmed when the average standard deviation of split frequencies fell below 0.01. The first 25% of sampled trees were discarded as burn-in, and a 50% majority-rule consensus tree was constructed to calculate posterior probabilities (PP).

### 2.5 Divergence time estimation

To estimate divergence times within 32 *Delphinium* species, we employed Bayesian analysis in BEAST v2.5.1 (Bouckaert et al., 2014) under a strict clock model. A strict clock was specifically selected over a relaxed clock because our dataset focuses on shallow, infrageneric relationships with low sequence divergence, where strict clocks are more appropriate to prevent over-parameterization (Ho and Duchêne, 2014). Given the absence of reliable fossil records within *Delphinium*, we utilized two secondary calibration strategies based on well-established divergence events of related taxa. Two secondary calibration points were employed to constrain the divergence times using lognormal distribution priors: the split between *T. alpinum* and *C. chinensis* (49 Ma, 95% HPD: 25.7-79.6 Ma) (Ramírez-Barahona et al., 2020), and the divergence between *T. alpinum* and *D. grandiflorum* (47 Ma, 95% HPD: 19.6-70.4 Ma) (Fior et al., 2013). The tree prior was set to a Yule speciation process. The MCMC analysis was run for 100 million generations, sampling parameters every 1,000 generations. The stationarity and convergence were assessed using Tracer v1.6 (Rambaut et al., 2014) to ensure that all parameters achieved effective sample sizes (ESS) greater than 200. After discarding the first 10% of trees as burn-in, a maximum clade credibility (MCC) tree with mean node ages and 95% highest posterior density (HPD) intervals was generated using TreeAnnotator v1.10.4. The resulting chronogram was visualized and annotated in FigTree v1.3.1 (Rambaut, 2009).

## 3 Results

### 3.1 Characteristics of *Delphinium* chloroplast genomes

**Figure 1:**
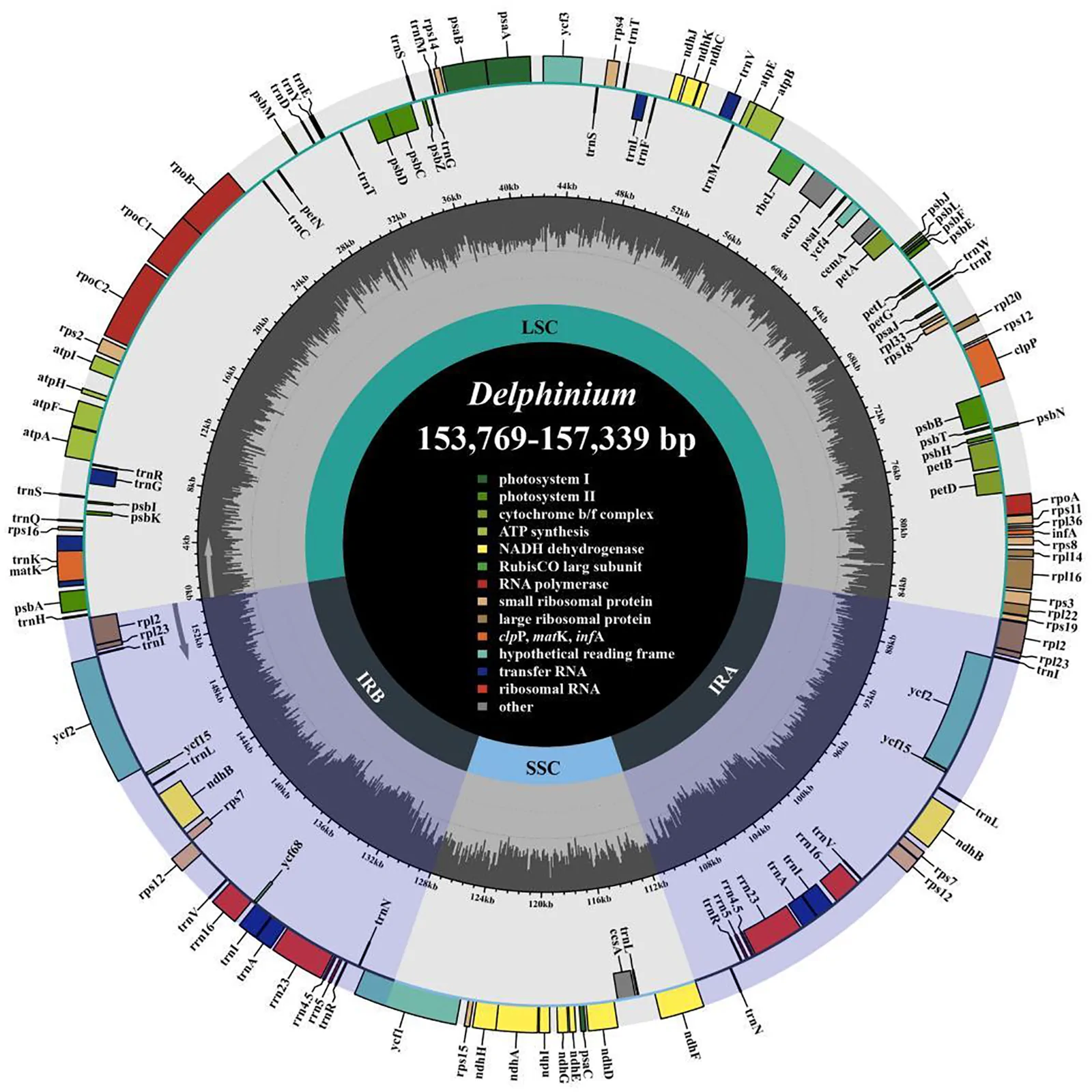
General chloroplast genome map of 32 *Delphinium* species. The specific genome sizes for each species are presented in **Table 1**.

The complete chloroplast genomes of the 32 *Delphinium* species analyzed in this study ranged in size from 153,769 bp in *D. pylzowii* to 157,339 bp in *D. grandiflorum* (**Table 1**). All assembled plastomes exhibited the typical quadripartite structure characteristic of angiosperms, consisting of a large single-copy (LSC) region and a small single-copy (SSC) region, separated by a pair of inverted repeat (IR) regions (**Table 1**; **Figure 1**). The LSC regions varied from 84,347 bp in *D. pylzowii* to 88,090 bp in *D. grandiflorum*, representing 54.85-55.99% of the total genome size. The SSC regions ranged from 16,047 bp in *D. candelabrum* to 17,252 bp in *D. anthriscifolium*, accounting for 10.42-11.12% of the total sequence. Additionally, the length of the IR exhibited significant variation, ranging from 25,977 bp in *D. anthriscifolium* to 26,594 bp in *D. naviculare*, *D. iliense*, and *D. winklerianum*, contributing 16.75-17.25% of the total genome length.

**Table 1:** Characteristics of newly sequenced plastomes.

Species	LSC length (bp)	IR length (bp)	SSC length (bp)	Plastome size (bp)	GC content (%)	Gene number	Protein coding genes	tRNA	rRNA	GenBank
★*D. albocoeruleum*	84,847	26,555	16,301	154,258	38.3%	131	85	37	8	PQ845753
★*D. densiflorum*	84,509	26,566	16,277	153,918	38.3%	131	85	37	8	PX644816
★*D. trichophorum*	84,750	26,548	16,271	154,117	38.3%	131	85	37	8	PX644817
*D. ceratophorum*	84,801	26,560	16,324	154,245	38.3%	128	83	37	8	MK253460
*D. anthriscifolium*	85,871	25,977	17,252	155,077	38.1%	128	83	37	8	MK253461
*D. candelabrum*	84,862	26,543	16,047	153,995	38.2%	130	83	37	8	MW246165
*D. maackianum*	85,055	26,564	16,301	154,484	38.1%	129	83	37	8	NC047293
*D. grandiflorum*	88,090	26,160	16,929	157,339	38.1%	128	80	36	8	NC049872
*D. brunonianum*	84,512	26,559	16,296	153,926	38.3%	131	86	37	8	NC051554
*D. yunnanense*	84,639	26,551	16,312	154,053	38.3%	130	83	37	8	MT759768
*D. caeruleum*	84,704	26,560	16,296	154,120	38.3%	130	86	36	8	PP234506
*D. tangkulaense*	84,890	26,555	16,316	154,316	38.2%	130	86	36	8	PP234555
*D. naviculare*	84,656	26,594	16,327	154,171	38.3%	128	83	37	8	OR263587
*D. aemulans*	84,809	26,561	16,314	154,245	38.3%	128	83	37	8	OR263583
*D. elatum*	84,780	26,561	16,317	154,219	38.3%	128	83	37	8	OR263584
*D. shawurense*	84,820	26,561	16,342	154,284	38.3%	128	83	37	8	OR263585
*D. iliense*	84,648	26,594	16,325	154,161	38.3%	128	83	37	8	OR263586
*D. mollifolium*	85,018	26,331	16,299	153,979	38.3%	128	83	37	8	OR263588
*D. winklerianum*	84,754	26,594	16,293	154,235	38.3%	128	83	37	8	OR263589
*D. beesianum*	84,795	26,554	16,325	154,228	38.2%	128	83	37	8	PX380655
*D. gyalanum*	84,872	26,558	16,332	154,320	38.3%	128	83	37	8	PX380660
*D. kamaonense*	84,464	26,552	16,281	153,849	38.3%	128	83	37	8	PX380665
*D. kingianum*	84,796	26,540	16,273	154,149	38.3%	128	83	37	8	PX380666
*D. pachycentrum*	84,752	26,554	16,287	154,147	38.3%	128	83	37	8	PX380667
*D. platyonychinum*	84,527	26,564	16,281	153,936	38.3%	128	83	37	8	PX380668
*D. pylzowii*	84,347	26,555	16,312	153,769	38.3%	128	83	37	8	PX380669
*D. spirocentrum*	84,776	26,539	16,283	154,137	38.3%	128	83	37	8	PX380670
*D. tatsienense*	84,785	26,554	16,329	154,222	38.2%	128	83	37	8	PX380671
*D. tenii*	84,746	26,554	16,291	154,145	38.3%	128	83	37	8	PX380672
*D. thibeticum*	84,779	26,554	16,290	154,177	38.3%	128	83	37	8	PX380673
*D. wangii*	84,776	26,561	16,312	154,210	38.3%	128	83	37	8	PX380676
*D. yuanum*	84,788	26,554	16,325	154,221	38.2%	128	83	37	8	PX380677

The three newly sequenced species in this study are indicated by red stars.

The overall gene content and organization remained highly conserved across the 32 *Delphinium* plastomes. We annotated 128–131 genes per genome, comprising 80–86 protein-coding genes, 36–37 tRNAs, and 8 rRNAs (**Table 1**). The overall GC content was similarly uniform among the species, ranging from 38.1% to 38.3%. Both the genomic architecture and GC content were remarkably conserved, aligning with patterns observed in other Ranunculaceae species. This high degree of conservation suggests a stable evolutionary trajectory and limited divergence in these fundamental genomic features within the family.

### 3.2 cpDNA markers for *Delphinium*

**Figure 2:**
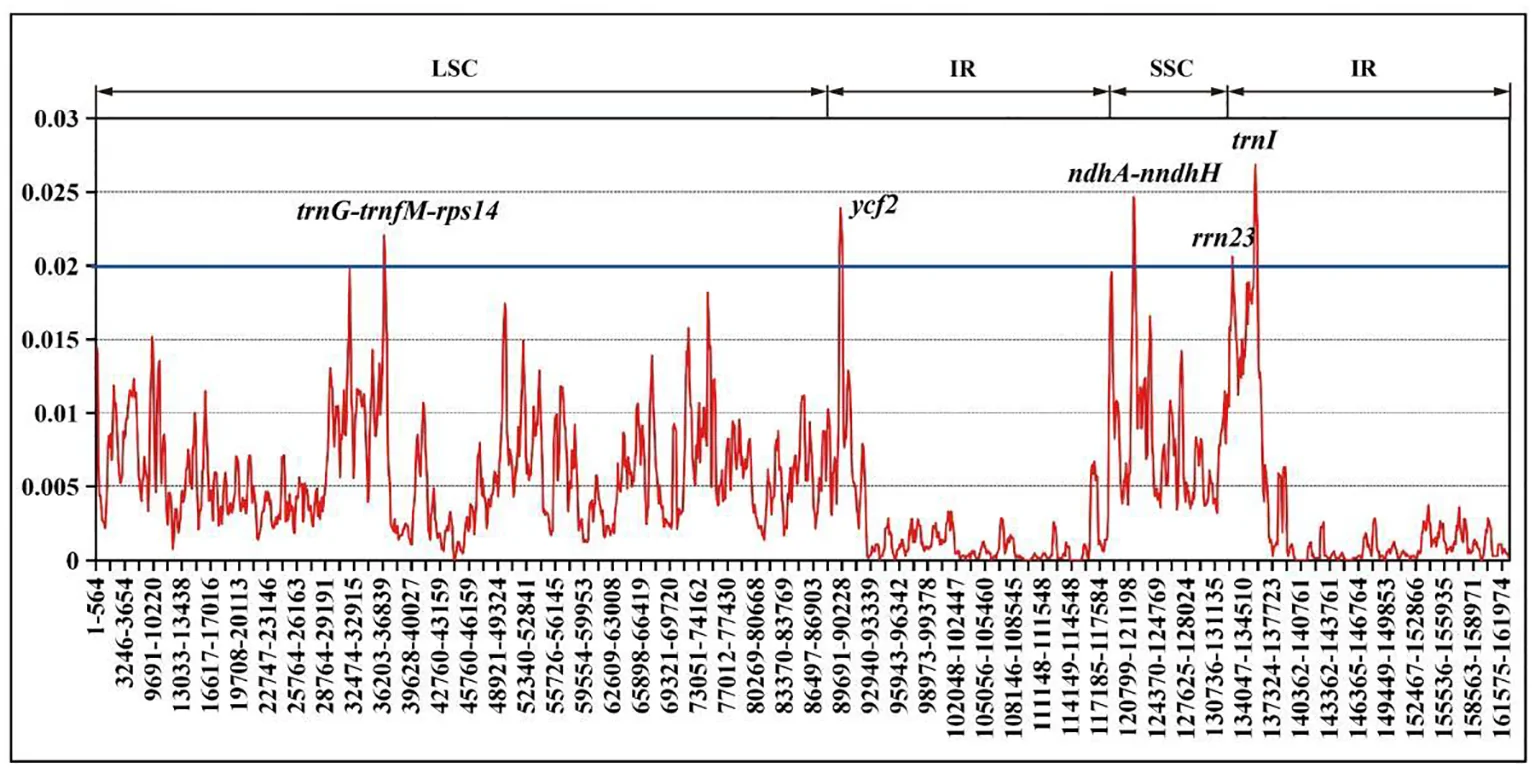
Sliding window analysis of nucleotide variability (*π*) across 32 complete chloroplast genome sequences of *Delphinium*.

To identify mutation hotspots and potential DNA barcodes for the genus *Delphinium*, we aligned the complete chloroplast genomes of 32 representative species. The resulting alignment spanned 162,923 bp in length. Within this dataset, a total of 7,681 polymorphic sites (4.71%) were identified, of which 2,514 (1.54%) were parsimony-informative sites and 5,048 (3.10%) were singleton variable sites. Interspecific sequence divergence, estimated by p-distance, ranged from 0.000 to 0.025. Nucleotide diversity (*π*) was assessed using a sliding window analysis with a window length of 400 bp and a step size of 100 bp (**Figure 2**). The average π value across the entire chloroplast genome was 0.00478.

Significant regional variation in nucleotide diversity was observed across the chloroplast genomes. Based on the sliding window analysis, narrow, localized variable windows (*π* > 0.02) were detected within or immediately adjacent to five highly conserved loci, including *trnG*-*trnfM*-*rps14*, *ycf2*, *ndhA*-*ndhH*, *rrn23*, and *trnI* (**Figure 2**). Among these, *trnG*-*trnfM*-*rps14* is situated in the LSC region, *ndhA*-*ndhH* resides in the SSC region, and the remaining three loci (*ycf2*, *rrn23*, and *trnI*) are located in the IR regions (**Figures 1**, **2**). However, when calculating the nucleotide diversity across the full length of these sequences, these loci exhibited extremely low overall variability. The average *π* value of these five markers was only 0.00183, with *rrn23* and *trnI* showing extreme conservation (*π* = 0.00022 and 0.00024, respectively). This overall variability is notably lower than the complete plastome average (*π* = 0.00478) and that of conventional plant DNA barcodes (e.g., *matK*, *rbcL*, and *trnH*-*psbA*, average *π* = 0.0043). Specifically, the conventional barcodes exhibited approximately 2.35 times higher variability than the overall sequences of these five loci. Therefore, rather than classifying these entire loci as “hypervariable hotspots,” our results indicate that they are fundamentally highly conserved regions that harbor narrow, localized variable windows (**Figure 2**; **Table 2**).

**Table 2:** Variability of the five loci containing localized hypervariable windows and the universal chloroplast DNA barcodes (*rbcL*, *matK* and *trnH*-*psbA*) in 32 *Delphiniu*m species.

Markers	Length	Variable sites		Parsimony informative sites		Singleton variable sites		Nucleotide diversity
		Numbers	%	Numbers	%	Numbers	%	
the entire genome	162,923	7,681	4.71	2,514	1.54	5,048	3.10	0.00478
*trnG-trnfM-rps14*	479	8	1.67	2	0.42	6	1.25	0.00204
*ycf2*	6,453	87	1.35	28	0.43	59	0.91	0.00142
*ndhA*-*ndhH*	3,355	193	5.75	56	1.67	136	4.05	0.00525
*rrn23*	2,810	4	0.14	2	0.07	2	0.07	0.00022
*trnI*	1,087	3	0.28	1	0.09	2	0.18	0.00024
*matK*	1,572	131	8.33	31	1.97	100	6.36	0.00801
*rbcL*	1,428	32	2.24	9	0.63	23	1.61	0.00194
*psbA-trnH*	1,137	36	3.17	9	0.79	27	2.37	0.00295

### 3.3 Simple sequence repeats and IR boundary analysis

**Figure 3:**
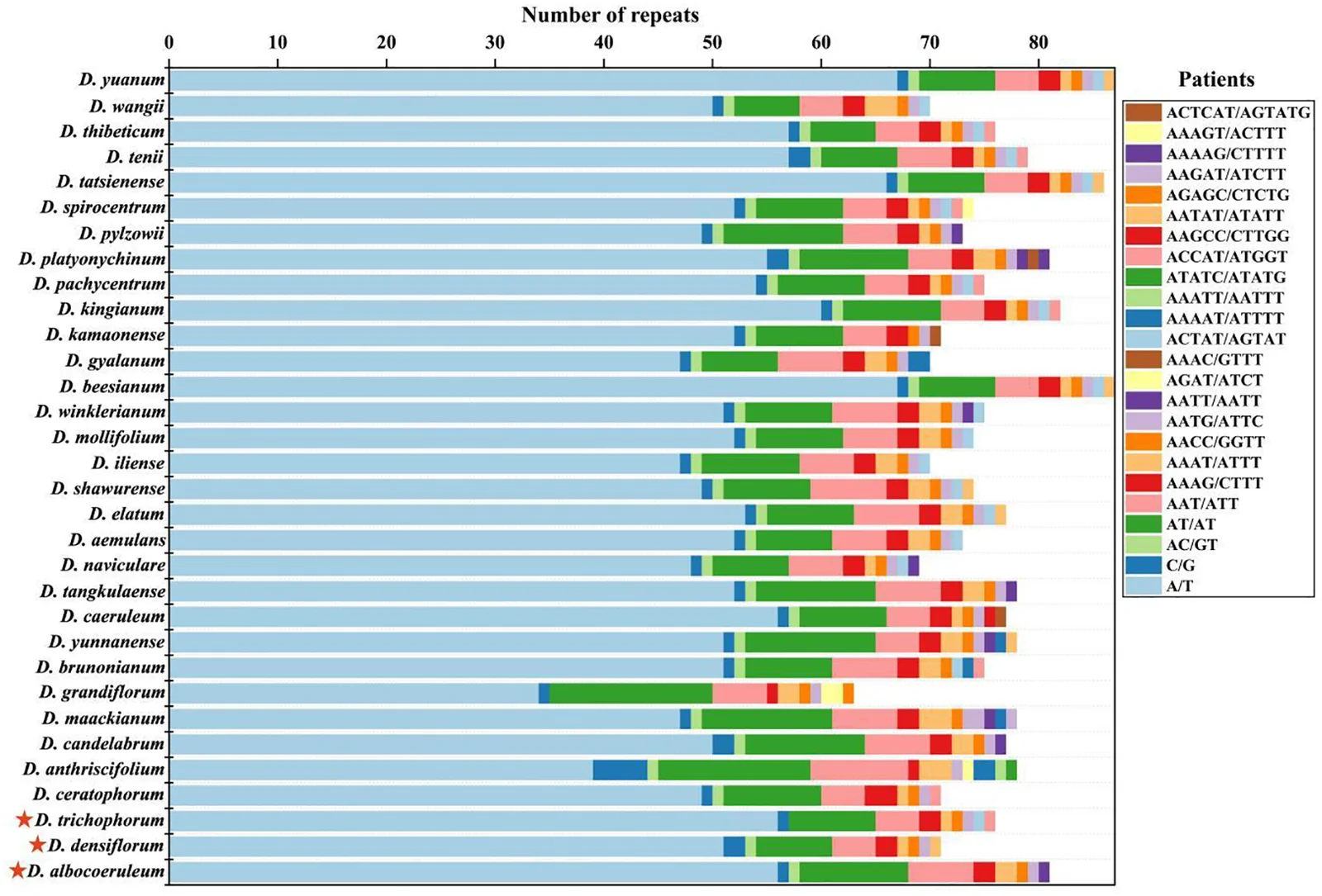
Abundance and categories distribution of simple sequence repeats in 32 *Delphinium* species. The three newly sequenced species in this study are indicated by red stars.

A total of 2,426 simple sequence repeat (SSR) loci were identified across the 32 examined *Delphinium* plastomes, with an average of 75.81 SSRs per genome (**Figure 3**). The abundance of SSRs varied among species, ranging from 63 in *D. grandiflorum* to 87 in *D. beesianum* and *D. yuanum*, indicating interspecific variation in microsatellite distribution. Based on repeat unit length, these SSRs were classified into six categories. Among them, mononucleotide repeats were the predominant type (70.77%, n = 1,717), followed by dinucleotide (12.82%, n = 311), tetranucleotide (8.53%, n = 207), and trinucleotide repeats (6.55%, n = 159). Pentanucleotide and hexanucleotide repeats were rare, collectively accounting for only 1.32% (n = 32) of the total. Notably, mononucleotide repeats predominantly consisted of A/T motifs, while dinucleotide repeats were dominated by AT/TA motifs. This composition pattern strongly aligns with the A/T bias typically observed in the plastid genomes of land plants.

**Figure 4:**
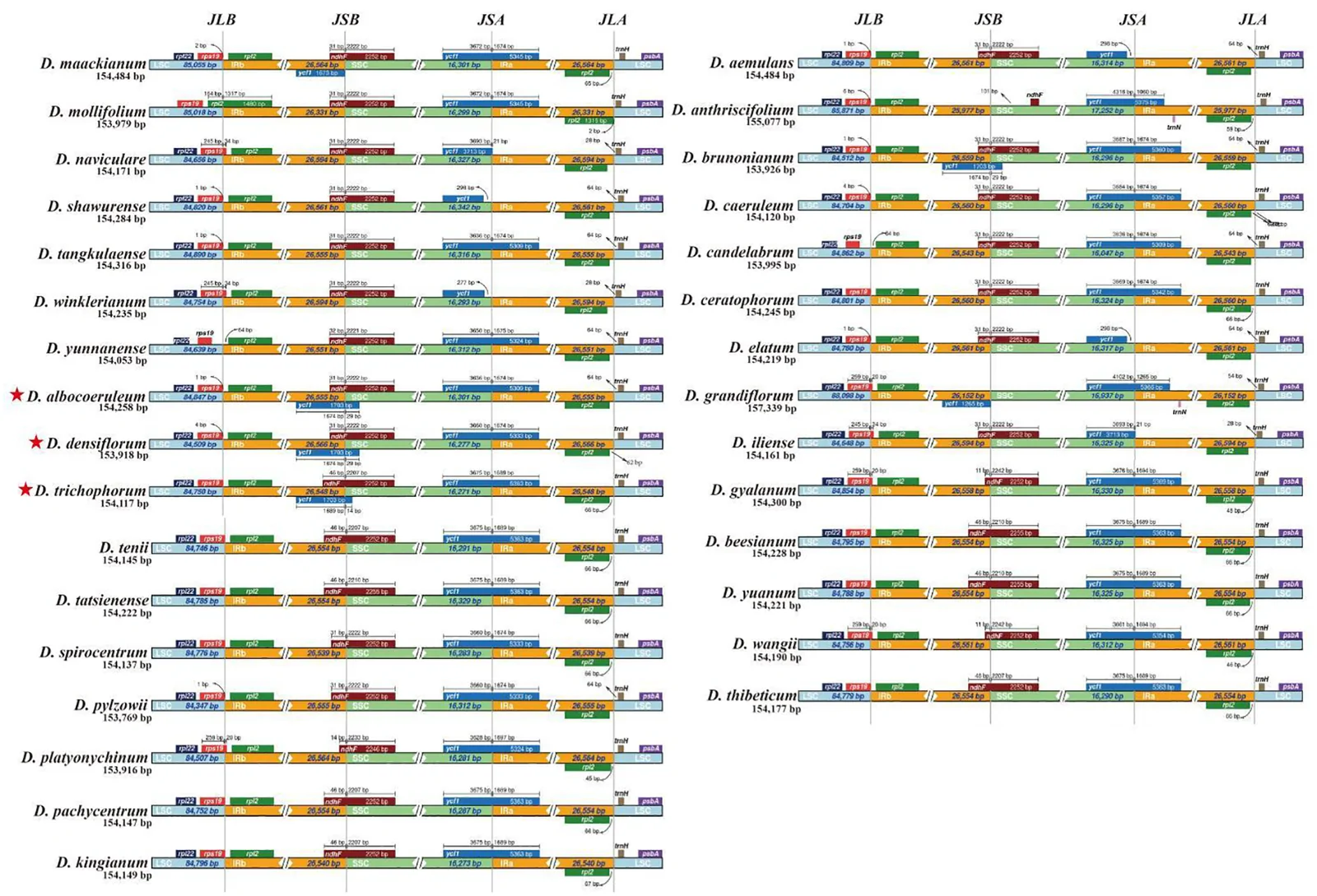
Schematic diagram of chloroplast genome boundary regions of 31 species. The chloroplast genome of *D. kamaonense* was not included in this analysis due to the absence of reliable IR boundary annotation from the public database. The three newly sequenced species in this study are indicated by red stars.

The dynamics of IR boundary expansion and contraction were visualized using *D. trichophorum* as a reference (**Figure 4**). Based on the structural relationships between key genes and the four junction sites (JLB: LSC/IRb; JSB: IRb/SSC; JSA: SSC/IRa; JLA: IRa/LSC), we classified the 31 *Delphinium* chloroplast genomes into five distinct boundary types (**Table 3**). Across all types, the JLA boundary exhibits high stability, with the *rpl2* and *trnH* genes consistently flanking this junction. The specific gene distributions for each type are detailed in **Table 3**.

**Table 3:** Structural characteristics and gene distributions across the four chloroplast IR boundaries in 31 *Delphinium* species.

Boundary type	Number of species	JLB (LSC/IRb)	JSB (IRb/SSC)	JSA (SSC/IRa)	JLA (IRa/LSC)
Type I (Predominant)	23	*rps19*, *rpl22*, and *rpl2* present. 1. *rps19* entirely in LSC in 17 species (*D. shawurense*, *D. tangkulaense*, *D. yunnanense*, *D. tenii*, *D. tatsienense*, *D. spirocentrum*, *D. pylzowii*, *D. pachycentrum*, *D. kingianum*, *D. aemulans*, *D. caeruleum*, *D. candelabrum*, *D. ceratophorum*, *D. elatum*, *D. beesianum*, *D. yuanum*, *D. thibeticum*). 2. *rps19* extends 1–34 bp into IRb in 6 species (*D. naviculare*, *D. winklerianum*, *D. platyonychinum*, *D. iliense*, *D. gyalanum*, *D. wangii*).	1. *ndhF* extends 11–46 bp into IRb of 22 species. 2. No *ycf1* pseudogene.	1. *ycf1* spans boundary, extending 21-1,697 bp into IRa in 19 species. 2. *ycf1* entirely in SSC in 4 species (*D. shawurense*, *D. winklerianum*, *D. aemulans*, and *D. elatum*).	Flanked by *rpl2* and *trnH* (highly conserved)
Type II	1	Same as Type I (*rps19* entirely in LSC)	1. *ndhF* entirely in SSC in *D. anthriscifolium*. 2. No *ycf1* pseudogene.	Same as Type I (*ycf1* spans boundary)	Same as Type I
Type III	5	Same as Type I (*rps19* entirely in LSC)	Presence of a duplicated *ycf1* pseudogene. 1. *ycf1* pseudogene in IRb in *D. maackianum*. 2. *ycf1* pseudogene extends 14–29 bp into SSC in 4 species (*D. albocoeruleum*, *D. densiflorum*, *D. trichophorum*, *D. brunonianum*).	Same as Type I (*ycf1* spans boundary)	Same as Type I
Type IV	1	Same as Type I (*rps19* extends into IRb)	1. *ndhF* absent from JSB in *D. grandiflorum*. 2. *ycf1* pseudogene extends 29 bp into SSC in *D. grandiflorum*.	Same as Type I (*ycf1* spans boundary)	Same as Type I
Type V	1	Only *rps19* and *rpl2* present in *D. mollifolium*; 5′ end of *rpl2* extends 164 bp into LSC (lacks *rpl22* at junction)	Same as Type I (*ndhF* extends into IRb; no *ycf1* pseudogene)	Same as Type I (*ycf1* spans boundary)	Same as Type I

Among these types, Type I represents the predominant pattern, characterized by the presence of *rps19*, *rpl22*, and *rpl2* at JLB, and the absence of a duplicated *ycf1* pseudogene at JSB. However, within Type I, variations are observed at the JSA boundary: the *ycf1* spans the boundary extending 21-1,697 bp into IRa in 19 species, whereas it is entirely confined to the SSC region in four species.

Type II is represented solely by *D. anthriscifolium*, in which *ndhF* is entirely located within the SSC region. Type III, comprising five species, is defined by the presence of a duplicated *ycf1* pseudogene at the JSB. Within this group, the pseudogene is entirely restricted to the IRb region in *D. maackianum*, whereas it extends 14–29 bp into the SSC region in the remaining four species.

Type IV is exclusively observed in *D. grandiflorum*. Although it shares the duplicated *ycf1* pseudogene with Type III, it is distinguished by the absence of *ndhF* at the JSB junction. Finally, Type V is unique to *D. mollifolium*, distinguished by the absence of *rpl22* at JLB and a 164-bp extension of the 5′ end of *rpl2* into the LSC region.

### 3.4 Phylogenetic analyses

**Figure 5:**
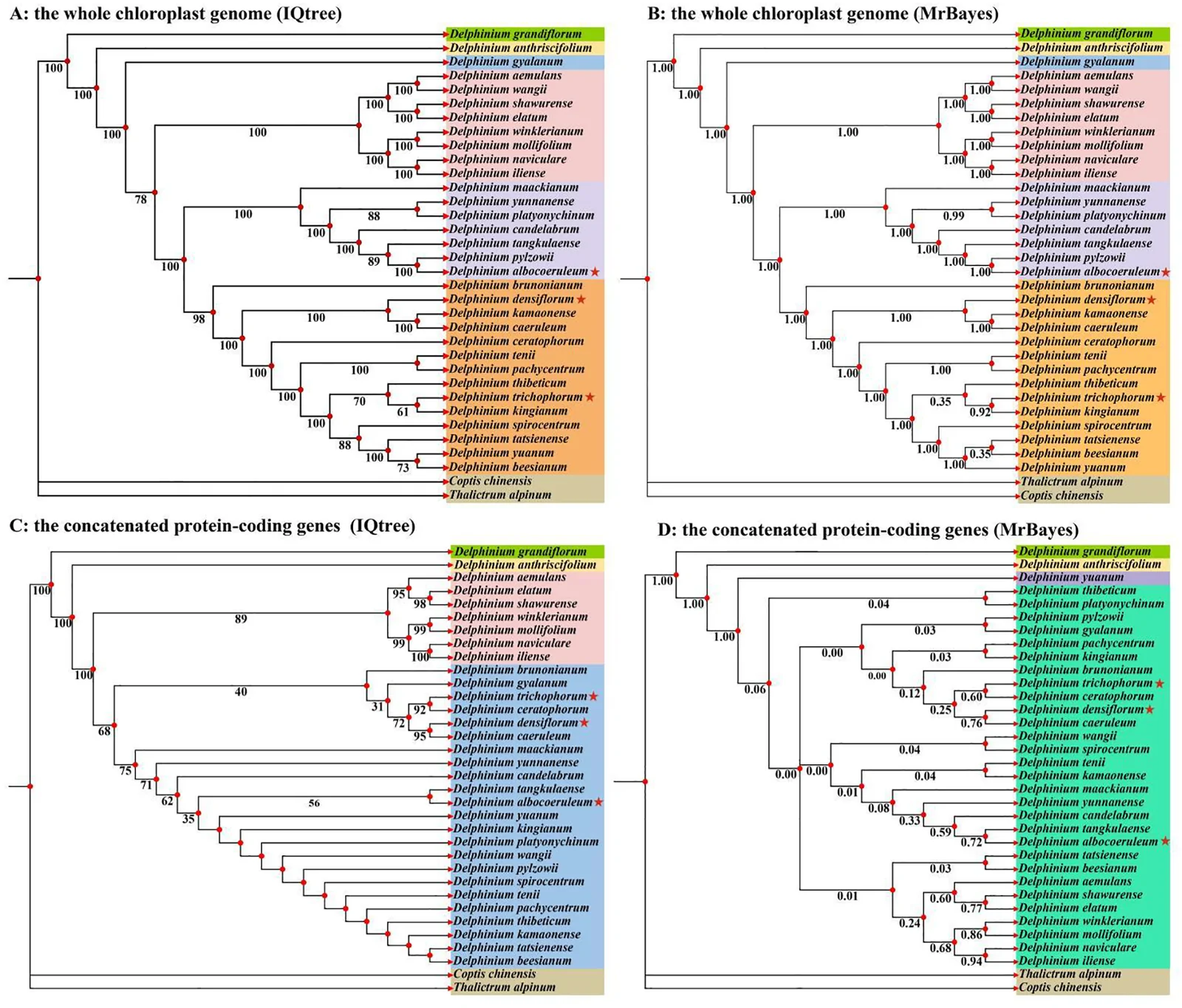
Molecular phylogeny of *Delphinium* from ML and BI analyses using two different datasets. **(A)**, Whole plastome ML tree; **(B)**, Whole plastome BI tree; **(C)**, PCGs ML tree; **(D)**, PCGs BI tree. Maximum likelihood bootstrap values (BS) and posterior probabilities (PP) are shown at nodes. Different colors represent different branches. The three newly sequenced species in this study are indicated by red stars.

To elucidate the phylogenetic relationships among the 32 *Delphinium* species, we performed phylogenetic reconstructions using two datasets and two inference methods. Our phylogenetic analyses consistently recovered *Delphinium* as a robust monophyletic group. The trees inferred from the whole chloroplast genome sequences revealed clear lineage divergence, partitioning the genus into six primary clades (Clades I-VI; **Figures 5A, B**). Clade I was identified as the earliest diverging lineage, comprising only *D. grandiflorum* (BS = 100, PP = 1.00). Clades II and III were each represented by a single species of *D. anthriscifolium* and *D. gyalanum*, respectively (BS = 100, PP = 1.00). Within the remaining group, Clade IV consisted of *D. aemulans*, *D. wangii*, *D. shawurense*, *D. elatum*, *D. winklerianum*, *D. mollifolium*, *D. naviculare*, and *D. iliense*, and formed a sister relationship with the subclade formed by Clades V and VI (BS = 78, PP = 1.00). Finally, Clade V and Clade VI were recovered as sister clades with high support (BS = 100, PP = 1.00), with the former comprising *D. maackianum*, *D. yunnanense*, *D. platyonychinum*, *D. candelabrum*, *D. tangkulaense*, and *D. albocoeruleum*, and the latter including the remaining 14 species.

In contrast, phylogenetic trees derived from concatenated protein-coding genes (PCGs) (**Figures 5C, D**) exhibited a distinct topology, resolving the taxa into only four primary clades (PCG-Clades I-IV). Notably, topological incongruence emerged between the IQ-TREE and BI analyses, particularly regarding the composition of PCG-Clades III and IV. Specifically, PCG-Clade I remained the earliest diverging lineage. The IQ-TREE (**Figure 5C**) and BI tree (**Figure 5D**) both recovered PCG-Clade II (*D. anthriscifolium*) as the second earliest diverging lineage. However, the composition of the remaining two clades differed between the two inference methods. In the IQ-TREE (**Figure 5C**), PCG-Clade III comprised *D. aemulans*, *D. elatum*, *D. shawurense*, *D. winklerianum*, *D. mollifolium*, *D. naviculare*, *and D. iliense*, while the remaining species formed PCG-Clade IV. In contrast, the BI tree (**Figure 5D**) recovered an alternative topology, in which PCG-Clade III contained only *D. yuanum*, and all other species were placed in PCG-Clade IV. Quantitative comparisons of clade support highlighted the superior resolution of the complete plastome dataset. The plastome phylogeny achieved maximum support (BS = 100, PP = 1.00) across all 32 internal nodes, with fully congruent topologies between ML and BI analyses (**Figures 5A, B**). In contrast, PCG-based trees yielded significantly lower support, with only 46.9% of nodes reaching BS ≥ 95 and 65.6% reaching PP ≥ 0.95 (**Figures 5C, D**), and exhibited topological incongruence between inference methods. Despite these localized discrepancies, the broader phylogenetic relationships among the species remained largely congruent across different datasets and inference methods. Collectively, these quantitative findings demonstrate that whole plastomes provide substantially higher nodal support and topological consistency than PCGs alone, establishing a robust foundation for future evolutionary analyses.

### 3.5 Divergence time estimate

**Figure 6:**
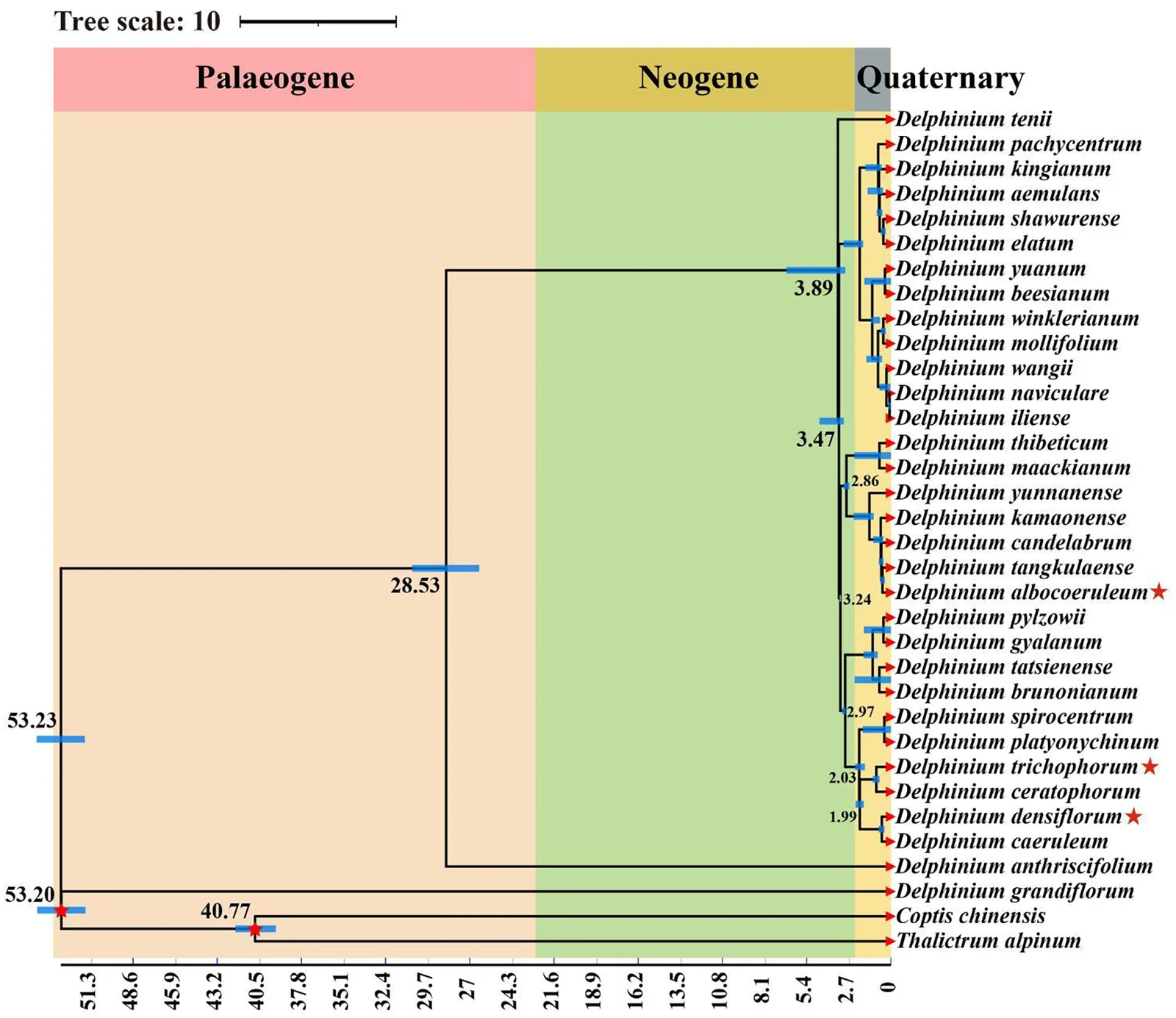
Maximum clade credibility (MCC) tree of 32 *Delphinium* species obtained from BEAST analysis. Mean divergence time estimates are shown with 95% highest posterior density (HPD; blue bars). Red pentagram indicates the two calibration points. The three newly sequenced species in this study are indicated by red stars.

The phylogeny reveals that the *Delphinium* lineage originated in the Eocene, approximately 53.23 Ma (95% HPD: 51.68-54.77 Ma; **Figure 6**), with the initial divergence between *D. grandiflorum* and the remaining species occurring shortly thereafter at 53.20 Ma (95% HPD: 51.64-54.77 Ma). Subsequently, a major lineage split occurred during the Oligocene at 28.53 Ma (95% HPD: 26.40-30.70 Ma), separating the basal lineages from the remaining species. More recent diversification events were concentrated in the Pliocene and Pleistocene. Specifically, the lineage leading to *Delphinium tenii* diverged at 3.89 Ma (95% HPD: 2.93-6.69 Ma), followed by a subsequent split at 3.47 Ma (95% HPD: 3.02-4.08 Ma) that gave rise to the major crown groups. Most intra-clade diversification events took place during the Pleistocene, a period characterized by profound climatic and geological shifts, such as the rapid uplift of the Qinghai-Tibet Plateau and the onset of Northern Hemisphere glaciations. These findings suggest that the evolutionary history of *Delphinium* has been shaped by a combination of ancient vicariance and recent climatic oscillations, which have collectively driven its extant diversity and complex biogeographic patterns.

## 4 Discussion

### 4.1 Comparative plastome analysis in 32 *Delphinium* species

Our comparative analysis of 32 *Delphinium* chloroplast genomes reveals a structurally conserved yet dynamically variable plastome that conforms to the canonical angiosperm quadripartite structure. Genome sizes ranged from 153,769 bp to 157,339 bp, and the inverted repeat (IR) regions exhibited length variations between 25,977 bp and 26,594 bp. This moderate variation in IR boundaries is likely driven by lineage-specific expansion or contraction events at the single-copy/IR junctions, a common mechanism of plastome size evolution in angiosperms (Zhu et al., 2016). Despite these boundary shifts, the consistent GC content (38.1-38.3%) and stable gene content (128–131 genes) across all examined species reflect strong structural conservation. This high degree of genomic stability closely mirrors the evolutionary patterns observed in its sister genus *Aconitum* (Xia et al., 2022) and other Ranunculaceae lineages such as *Clematis* (Wei et al., 2022), indicating a stable evolutionary trajectory for fundamental plastid features within the family.

The overall nucleotide diversity (*π* = 0.00478) and the proportion of polymorphic sites (4.71%) in *Delphinium* are similar to the patterns documented in *Rhododendron* (Li et al., 2023), reflecting moderate levels of interspecific divergence. Notably, sequence divergence was unevenly distributed across the genomic regions. The SSC region exhibited the highest diversity (*π* = 0.00525), followed by the LSC region (*π* = 0.00204), while the IR regions were the most conserved (*π* = 0.00063). Although sliding-window analysis detected narrow, localized variable windows (π > 0.02) within or adjacent to five specific loci (*trnG*-*trnfM*-*rps14*, *ycf2*, *ndhA*-*ndhH*, *rrn23*, and *trnI*), the overall sequences of these markers exhibited an exceptionally low average nucleotide diversity (*π* = 0.00183), indicating that they are highly conserved within the genus. It is noteworthy that three of these loci (*ycf2*, *rrn23*, and *trnI*) are located within the IR regions. The extreme conservation of these IR-residing loci is likely maintained by copy-dependent repair mechanisms (gene conversion), which significantly constrain mutation rates in the inverted repeats of land plants (Zhu et al., 2016). The elevated variability in the SSC is particularly significant given its enrichment with genes encoding photosystem I and the NADH dehydrogenase complex (*ndh* genes). This pattern may reflect adaptive evolution related to photosynthesis and energy metabolism (Liang et al., 2020). This distribution contrasts sharply with the pattern observed in *Cypripedium*, where hotspots are predominantly concentrated in the LSC region (Zhang et al., 2022). This divergence highlights the distinct evolutionary trajectories of plastid genomes across different lineages (Zhao et al., 2020). In contrast, conventional plant DNA barcodes (e.g., *matK*, *rbcL*, and *trnH*-*psbA*) exhibited approximately 2.35 times higher variability (*π* = 0.0043). This suggests that while the newly identified conserved loci may be useful for higher-level phylogenetic studies (e.g., family or order level), the conventional barcodes or SSC-derived hypervariable regions remain more suitable for species-level discrimination (Dong et al., 2015).

The distribution and composition of SSRs in chloroplast genomes provide critical insights into plastome evolution and offer valuable markers for population genetics (Provan et al., 2001). Across the 32 *Delphinium* plastomes, we identified an average of 75.81 SSRs per genome. The observed interspecific variation in SSR abundance, ranging from 63 to 87, reflects dynamic mutational processes—such as slipped-strand mispairing—during the evolutionary history of the genus (Levinson and Gutman, 1987). Consistent with typical angiosperm plastomes, mononucleotide repeats dominated the SSR profile (70.77%) and were predominantly composed of A/T motifs. Similarly, dinucleotide repeats exhibited a strong bias toward AT/TA motifs. This pronounced A/T richness is a ubiquitous feature of land plant plastomes, generally attributed to the lower thermostability of A-T bonds, which facilitates strand separation during plastid DNA replication (Kuang et al., 2011). This compositional bias closely mirrors the SSR profiles of related Ranunculaceae genera, such as *Aconitum* (Park et al., 2017), indicating a conserved mechanism of microsatellite evolution within the family. Despite the conserved motif composition, the quantitative variation of SSRs among *Delphinium* species underscores their practical utility. Given their uniparental inheritance and high intraspecific polymorphism, the chloroplast SSRs (cpSSRs) are regarded as a valuable genomic resource. They offer highly informative molecular markers for future studies on population genetics, phylogeography, and species delimitation in *Delphinium*, particularly for resolving closely related taxa that remain indistinguishable using conventional DNA barcodes (Hollingsworth et al., 2011). In this context, the comprehensive catalog of cpSSR loci generated in this study serves as a critical foundational resource. However, it is important to note that their definitive diagnostic value requires further empirical validation. Future studies must undertake targeted primer design, rigorous polymorphism screening across extensive population-level samplings, and sequencing validation to mitigate potential size homoplasy artifacts, thereby confirming their species-specificity and reliability.

Comparative analysis of the IR/SC boundary regions identified five distinct structural types, among which Type I was the predominant pattern. The expansion and contraction of the IR/SSC junctions, particularly involving the *ycf1* and *ndhF* genes, reflect a dynamic evolutionary history, a phenomenon similarly reported in *Pedicularis* (Wu et al., 2021) and *Rhodiola* (Tao et al., 2023). Furthermore, due to their relatively high nucleotide substitution rates and adequate sequence lengths, these boundary-spanning genes (*ycf1* and *ndhF*) provide valuable phylogenetic signals and have been widely utilized in resolving complex interspecific relationships (Dong et al., 2015). Although these structural variations at the boundaries do not alter the overall phylogenetic topology, they highlight the dynamic nature of plastome evolution within an otherwise highly conserved genomic framework.

### 4.2 Phylogenetic relationships of 32 *Delphinium* species

Establishing a robust phylogenetic framework for *Delphinium* is critical for understanding its diversification and biogeography. Our results demonstrate that whole chloroplast genome sequences yield higher phylogenetic resolution and stronger clade support than concatenated protein-coding genes (PCGs) in reconstructing the infrageneric relationships. This enhanced resolving power is primarily attributed to the inclusion of abundant, highly variable non-coding regions (e.g., introns and intergenic spacers), which typically exhibit significantly higher nucleotide substitution rates than conserved PCGs in angiosperms (Shaw et al., 2007). Because *Delphinium* has undergone recent and rapid evolutionary radiation, its interspecific sequence divergence remains relatively low (Song et al., 2024). Consequently, the phylogenetically informative sites (PIS) provided by PCGs alone are insufficient to fully resolve its complex phylogeny. This limitation manifests as lower node support in PCG-based trees and topological incongruence between maximum likelihood (IQ-TREE) and Bayesian inference (BI) analyses. In contrast, the whole chloroplast genome provides a wealth of polymorphic sites, effectively mitigating stochastic errors caused by a paucity of phylogenetic signal. These findings are consistent with recent phylogenomic studies within Ranunculaceae (Luo et al., 2026), confirming that plastome-scale data outperform limited marker sets in resolving rapid radiation events.

In this study, the plastome-based tree resolves *D. grandiflorum* (Clade I), *D. anthriscifolium* (Clade II), and *D. gyalanum* (Clade III) as the earliest-diverging lineages. These basal taxa exhibit remarkable morphological and developmental distinctiveness, likely retaining ancestral character states. As the first diverging lineage, *D. grandiflorum* is characterized by large, wide-open, bright blue flowers (with perianth segments 13–25 mm long and spurs 12–23 mm long), deeply dissected leaves with narrowly linear lobes, and a relatively simple racemose inflorescence (Wang and Warnock, 2001). Notably, this species lacks the highly specialized resupinate petals frequently observed in other lineages. Furthermore, its floral zygomorphy is established late in development (post-organogenesis), suggesting the retention of a more ancestral developmental program (Jabbour et al., 2009). Clade II consists exclusively of *D. anthriscifolium*, whose unique morphological characteristics further corroborate its isolated phylogenetic position. Unlike the majority of perennial species within the genus, this taxon is an annual herb featuring relatively small flowers and twice or thrice pinnately compound leaves that resemble those of the genus *Anthriscus* (Wang and Warnock, 2001). Developmental studies have revealed that the lateral petals of *D. anthriscifolium* undergo a 180° rotation (petal resupination) prior to anthesis—a phenomenon reported for the first time in Ranunculaceae and extremely rare within the family (Zhang et al., 2021). This unique floral ontogenetic trait likely represents an adaptive innovation for specific pollinator interactions, further justifying the recognition of this lineage as a distinct clade. *D. gyalanum* (Clade III) occupies a similarly isolated phylogenetic position. Although detailed developmental studies on this species remain limited, the basal placement of Clades I–III in the plastome tree collectively suggests that these taxa represent ancient lineages that diverged prior to the main evolutionary radiation of *Delphinium*.

Furthermore, phylogenetic analyses recovered Clade V and Clade VI as sister clades with maximum statistical support (BS = 100, PP = 1.00). Together, these two clades comprise the vast majority of the sampled species in this study, with Clade V containing six species (including *D. maackianum*) and Clade VI encompassing 14 species. This sister relationship, coupled with the remarkable species richness, suggests that this lineage underwent a relatively rapid evolutionary radiation (Jabbour and Renner, 2012). The high species diversity observed in Clade VI, in particular, likely reflects a successful adaptation to the complex geographic and ecological environments of the Sino-Himalayan region, a renowned biodiversity hotspot (Myers et al., 2000). Geological uplifts and climatic changes in this region during the Late Miocene provided abundant ecological niches for the core *Delphinium* lineages, thereby triggering their rapid speciation across diverse habitats (Song et al., 2024; Jabbour and Renner, 2012).

Despite the robust plastome-based framework, the PCG tree exhibits topological incongruence regarding the composition of PCG-Clades III and IV. This discrepancy is likely not only a consequence of the limited PIS in recently radiated lineages, but may also reflect historical events such as incomplete lineage sorting (ILS) or ancient hybridization (Chartier et al., 2016). Given its uniparental inheritance, the chloroplast genome alone cannot fully capture reticulate evolutionary histories, which frequently leads to severe plastid-nuclear phylogenetic conflicts in the presence of ILS or hybridization (Linder and Rieseberg, 2004). Therefore, subsequent studies must integrate nuclear genomic data to further unravel the complex speciation mechanisms of *Delphinium*.

### 4.3 Divergence time of 32 *Delphinium* species

Our molecular dating results, based on concatenated protein-coding genes and a strict clock model, indicate that the crown group of *Delphinium* originated in the early Eocene (ca. 53.23 Ma). Although this estimate is older than the early Oligocene origin (~32.3-33.3 Ma) proposed for the genus by previous tribe-level studies (Jabbour and Renner, 2012; Luo et al., 2026), such discrepancies are expected when comparing genus-level and tribe-level studies. Their tribe-level analyses of Delphinieae included sparse internal sampling of *Delphinium*, whereas our denser genus-level sampling likely captured earlier diverging lineages, naturally yielding an older crown age estimate (Linder et al., 2005). Beyond sampling density, previous dating results often relied on a limited number of markers (e.g., ITS and *trnL*-*F*), whereas the concatenated protein-coding genes employed in our study provide richer genetic information, effectively mitigating the underestimation of branch lengths at deep phylogenetic nodes. Differences in dating models (e.g., strict versus relaxed clocks) and calibration strategies may also contribute to this temporal discrepancy. This Eocene origin aligns well with the ancient evolutionary history of the family Ranunculaceae and its extant biogeographic patterns (Emadzade and Hörandl, 2011). Consequently, subsequent studies will integrate reliable Ranunculaceae macrofossils as direct calibration points, in conjunction with whole-genome data and relaxed clock models, to further cross-validate the early divergence time of this genus.

Following its early establishment in the Eocene, our results reveal that *Delphinium* underwent a major lineage divergence during the Oligocene (ca. 28.53 Ma), which separated the basal lineages from the core clades. This divergence event aligns with the prolonged global cooling and environmental shifts that followed the Eocene-Oligocene Transition (EOT) (Zachos et al., 2001). Driven by a sharp decline in global temperatures and intensified aridification in high-latitude and inland regions, the previously continuous suitable habitats of early *Delphinium* ancestors likely experienced severe contraction and fragmentation (Jabbour and Renner, 2012). Such climate-driven habitat fragmentation triggered strong vicariance, forcing isolated populations to evolve independently within their respective refugia (Xiang et al., 2017). Consequently, this process shaped the first major phylogenetic branching in the evolutionary history of the genus.

While these ancient climatic shifts established the basal phylogenetic framework of the genus, the more recent and exPLoSive diversification events within *Delphinium* were highly concentrated during the Pliocene and Pleistocene (e.g., the divergence of the *D. tenii* lineage at 3.89 Ma and the rapid radiation of the core crown group at 3.47 Ma). The surge in speciation rates during this period strongly reflects the ecological impacts of the rapid uplift of the QTP and the Hengduan Mountains (Favre et al., 2015; Xing and Ree, 2017). Intense orogeny generated highly heterogeneous topographies and novel alpine habitats, providing abundant ecological niches for *Delphinium* and driving robust adaptive radiation and allopatric speciation (Ding et al., 2025). Furthermore, recurrent Northern Hemisphere glaciations during the Pleistocene subjected the distribution ranges of alpine plants to repeated cycles of expansion and contraction (Hewitt, 2000). This “species pump” effect not only facilitated gene flow among populations followed by subsequent isolation and divergence, but also ultimately shaped the exceptionally high extant species diversity and complex biogeographic patterns of *Delphinium* (Ding et al., 2020; Cao et al., 2025).

## Data Availability

The datasets presented in this study can be found in online repositories. The names of the repository/repositories and accession number(s) can be found below: https://www.ncbi.nlm.nih.gov/, PQ845753 https://www.ncbi.nlm.nih.gov/, PX644816 https://www.ncbi.nlm.nih.gov/, PX644817.
